# DNA-Guided Metallization of Nanomaterials and Their Biomedical Applications

**DOI:** 10.3390/molecules28093922

**Published:** 2023-05-06

**Authors:** Ke Li, Yanfei Liu, Beibei Lou, Yifu Tan, Liwei Chen, Zhenbao Liu

**Affiliations:** 1 Department of Pharmaceutics, Xiangya School of Pharmaceutical Sciences, Central South University, Changsha 410013, China; 2 Department of Pharmaceutical Engineering, College of Chemistry and Chemical Engineering, Central South University, Changsha 410083, China; 3 Molecular Imaging Research Center of Central South University, Changsha 410008, China

**Keywords:** DNA nanotechnology, nanomaterials, metallization, morphology, biomedical application

## Abstract

Precise control of the structure of metallic nanomaterials is critical for the advancement of nanobiotechnology. As DNA (deoxyribonucleic acid) can readily modify various moieties, such as sulfhydryl, carboxyl, and amino groups, using DNA as a directing ligand to modulate the morphology of nanomaterials is a promising strategy. In this review, we focus on the use of DNA as a template to control the morphology of metallic nanoparticles and their biomedical applications, discuss the use of DNA for the metallization of gold and silver, explore the factors that influence the process, and outline its biomedical applications. This review aims to provide valuable insights into the DNA-guided growth of nanomaterials. The challenges and future directions are also discussed.

## 1. Introduction

The use of nanomaterials has brought significant advances in various fields, including biomedical applications. However, the synthesis of these materials often involves complex and expensive processes. Metallic nanomaterials are typically synthesized by wet chemistry or photolithography. However, metallic materials with complex shapes cannot be prepared by traditional wet chemical methods [1]. Although photolithography can control the structure of the material, it requires expensive production equipment and is limited by the optical diffraction limit [2,3]. DNA(deoxyribonucleic acid)-guided metallization of nanomaterials has emerged as a promising technique to overcome these challenges. DNA-guided metallization of nanomaterials refers to the process of using DNA molecules as templates to guide the synthesis and assembly of metallic nanoparticles [4,5]. This technique offers a simple, cost-effective, and efficient way to produce a wide range of nanomaterials with tailored shapes and sizes for various applications. The use of DNA as a template for metallization provides a high level of control over the properties of the resulting nanomaterials. Notably, DNA has been introduced during the growth phase of metal nanoparticles to control and regulate the morphology of the particles [6]. In this context, we review the latest developments in DNA-guided metallization of nanomaterials and their biomedical applications, as well as outline future research directions to enhance the potential of this technology.

In 1998, Braun et al. [7] proposed the use of DNA as a template to guide the growth of metallic materials. However, in the early stages of development, metallization was usually uniformly deposited along the DNA scaffold, compromising its biorecognition and addressability. To address these issues, researchers have developed several new strategies. For instance, DNA can be specifically labeled with an aldehyde group (e.g., glutaraldehyde), which acts as a local reductant on DNA and can be programmed to direct the metallization process only on selected DNA strands [8,9]. Moreover, the difference in affinity between DNA bases and metals can be exploited to achieve sequence-selective metallization [10]. This is because various nucleic acid bases have different affinities for metallic materials. Generally, DNA-directed metal particle synthesis involves two steps: first, metal nanoseeds bind to DNA as nucleation sites for selective metal deposition, and then metal atoms will form a continuous metal structure along the shape of DNA [4]. For gold nanomaterials, the relative adsorption affinity of DNA bases is adenine (A) > cytosine (C) ≥ guanine (G) > thymine (T) [11]. In the case of silver nanomaterials, G and C bases exhibit higher affinities for silver ions, and guanine has the highest oxidation potential among the nucleic acid bases, which leads to the specific binding of metal ions to GC-rich DNA. Upon oxidation and binding to DNA, silver atoms can gain electrons from the reduced guanine base. A series of successive cycles of oxidation and the transfer of Ag atoms from nanoparticle to DNA results in the localization of atoms at specific sites along the DNA, or the formation of a small number of atomic silver clusters near the sites of particle binding on poly(dG)-poly(dC) molecules [10].

Continuous development has shown that DNA sequence combinations can control the different morphologies of nanoparticles during their growth, and that these effects can be synergistic or competitive. The DNA used to guide the growth of nanomaterials is stable and retains its biological recognition capabilities [12]. As a result, the synthesis of nanocrystals with controlled three-dimensional structures using DNA is both feasible and appealing. The approach using DNA enables the solution-based synthesis of nanocrystals with controlled three-dimensional structures in the desired orientation, and extends the current tools available for the design and synthesis of functionally rich nanomaterials for future translational biotechnology.

The unique properties of these DNA-guided metal nanomaterials have led to their exploration in a range of biomedical applications. Metal nanoparticles have unique optical, electronic and magnetic properties [13]. DNA-directed metallization of nanomaterials can combine the unique physicochemical properties of metal nanomaterials with the biological functions of DNA for biomedical applications, especially in biodetection, biosensing, therapy or medical imaging.

In this review, we provide an overview of the current state of research in this field, with a focus on the synthesis and characterization of DNA-guided metal nanomaterials, as well as their biomedical applications. We highlight the progress made in the development of novel synthetic strategies and the optimization of existing methods to improve the control and precision of the resulting nanostructures. Furthermore, we discuss the potential of DNA-guided metal nanomaterials in various biomedical applications, including drug delivery, imaging, and sensing. We describe the strategies employed to enhance the biocompatibility and stability of these nanomaterials. Lastly, we outline the challenges that remain in the development and application of DNA-guided metal nanomaterials in biomedicine, such as their scalability for large-scale production, long-term stability, and precise control over their morphology. We also discuss the potential avenues for future research, such as the exploration of new biomedical applications and the integration of multiple functions into a single DNA-guided metal nanomaterial. Overall, DNA-guided metallization of nanomaterials holds significant promise in the field of biomedicine, and this review provides a comprehensive overview of the current state of research and the future directions of this exciting area.

## 2. Strategies of DNA Functionalized Nanoseeds

The formation of metal nucleation sites depends on the binding of metal ions or complexes to DNA and their subsequent reduction to form metal clusters, or on the binding of small metal particles to DNA.

In the DNA-directed assembly of nanoparticles, chemical bonds are commonly used to connect DNA to nanoparticles, such as the Au-S bond commonly used in gold nanoparticles [14,15,16]. Silver nanoparticles are commonly modified with DNA using disulfide, lipoic acid, or cyclic disulfide bonds [17,18,19]. Compared to Au-S, Ag-S chemical bonding is less stable, and, hence, SH-DNA modification of silver nanoparticles is not commonly used. Chemically bonded covalent modifications possess better specificity, but they are cumbersome to pre-modify. However, when DNA is used to guide the growth of nanomaterials, it is more common to use electrostatic or ligand interactions to bind DNA to metallic nanomaterials [20,21,22,23,24,25,26]. This is because the process of metallization often occurs in a solution where DNA is added to a solution containing metal ions for co-incubation [27]. The positively charged ions are attracted to the negatively charged phosphate backbone in the DNA backbone, and the DNA catalyzes their reduction, leading to the formation of metal clusters along the DNA [9]. These small clusters tend to grow into nanoparticles, and DNA strands covered with negative charges on the surface prevent aggregation. Reduction methods include chemical reduction, photochemical reduction [28,29], and electrochemical deposition [30,31]. Additionally, polymorphic DNA chains, such as polyA chains, can be rapidly adsorbed on the surface of gold nanoparticles [32,33], while silver can be specifically ligated to C bases (Ag-C) [34]. In other words, DNA can be modified on the surface of silver nanoseeds by intrinsic Ag-C ligands. The strong Ag-C ligand not only makes Ag-DNA couples easy to form, but also shows good stability under high ionic strength and high-temperature conditions [35,36].

## 3. DNA as Director for Nanomaterials Metallization

In this section, we mainly focus on the use of DNA to guide the metallization of metallic nanomaterials. So far, the main discussion has been on the functionalization of nanomaterials using DNA after their synthesis, where the morphology of the nanomaterials is already determined and DNA cannot influence it. The method of seed-mediated synthesis using DNA as a director can be used to prepare nanomaterials with controlled morphology [6]. The success of this approach is mainly based on the fact that different bases of DNA have different affinities for the surface of nanomaterials, resulting in different growth rates and morphologies. For instance, taking gold nanomaterials as an example, the adsorption affinities of different bases are as follows: A > C ≥ G > T [11]. Moreover, DNA can be easily modified with various groups such as thiol groups, carboxyl groups, and amino groups, which further extends the overgrowth method to regulate the morphology of nanoparticles using nanoparticles as seeds and DNA as a guiding agent.

DNA metallization can be divided into two main steps: assembling the DNA template and organizing the nanoparticles [37,38]. The first step involves activation, in which the negatively charged DNA binds to cations or nanoparticles through coordination or electrostatic interactions. The next step is reduction, in which the reducing agent converts the metal cations into initial clusters. The commonly used reducing agents in this process include chemical reagents (e.g., NaBH_4_, ascorbic acid), UV light, and electricity [39,40,41]. Finally, in the growth step, unbound or newly introduced ions/nanoparticles are further reduced or deposited on the previous metal cluster sites [42]. The shape of the generated nanostructures is mainly influenced by two factors: the initial shape of the nano-seed and the sequence and secondary structure of the DNA. The size, orientation, and anisotropy of the crystals can be regulated by controlling nanoparticle concentration, solution ionic strength, and DNA grafting density on the particles and surface.

In addition, metal nanocrystals with tunable surface plasmon resonance (SPR) can be obtained by using template molecules to guide the precise growth of metal atoms along a designed template [43,44,45,46,47,48,49]. Proper metallization of DNA can improve its electrical conductivity while maintaining the geometric features of DNA nanostructures. This imparts magnetic and optical properties to DNA and expands its potential applications. DNA-guided metal nanocrystals can be precisely controlled relative to the SPR peak position, and their optical properties can be further enhanced by controlling their morphology [50]. Currently, gold is one of the most widely investigated metal nanocrystals. Different DNA templates produce nanocrystals with various morphologies and specific SPRs by regulating the growth of gold along the DNA backbone. As the number of metal branches guided by the template DNA increases, the surface of the synthesized nanocrystals becomes rougher, enhancing the plasmon resonance between the nanoparticle core and the branches, and leading to the maximum absorption redshift peak. Moreover, different sizes of metal branches due to different DNA structures also cause the maximum absorption wavelength to change [49,51,52]. Potential DNA templates have different secondary structures, such as single-stranded (ss), double-stranded (ds), hairpin (hp), and triple-junction arms (ta). Researchers have successfully synthesized several shapes of DNA-directed nanostructures, including nanoflowers, nanopolygons, nanocaps with nanobridges, and sea cucumber-like structures [53]. Some examples related to these structures are listed in Table 1.

### 3.1. ssDNA

During crystal growth, different DNA sequences can influence the morphology of the generated gold nanoparticles. DNA with a chain-like structure can guide the deposition of reduced gold on gold nano-seeds and direct the nanoparticles to form different shapes [11,60,61]. Gold nano-seeds and DNA templates are essential cores for the formation of AuNCs of different morphologies.

**Figure 1:**
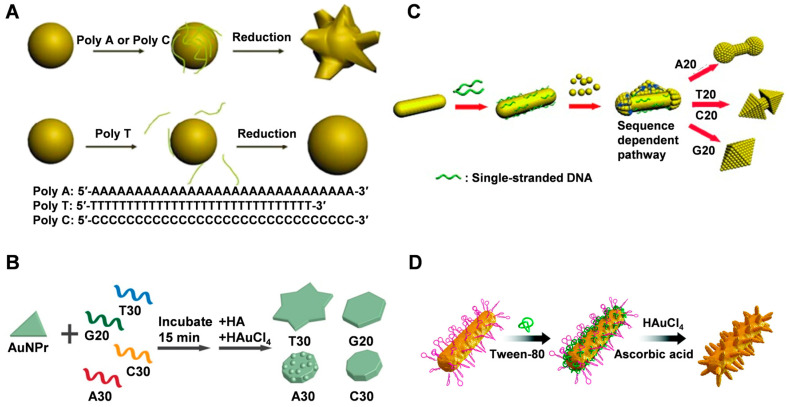
(**A**) Schematic illustration of the DNA mediated shape control of gold nanoparticles. Reproduced with permission [60]. Copyright 2010, American Chemical Society. (**B**) DNA-encoded growth of Au nanoprism seeds into four shapes using the four different DNA strands: T30, G20, C30, and A30. Reproduced with permission [11]. Copyright 2015, American Chemical Society. (**C**) A proposed mechanism of overgrowth of AuNRs by homooligomeric DNA with different sequences. Reproduced with permission [61]. Copyright 2015, Wiley-VCH. (**D**) Schematic of the synthesis of trepang-like AuNCs. Reproduced with permission [41]. Copyright 2019, The Royal Society of Chemistry.

Thiol-modified single-stranded DNA and poly A/C/G/T DNA chains have been used to guide the synthesis of metal nanocrystals with specific morphologies by utilizing the affinity of different bases for the surface of core metal nanoparticles. Wang et al. [60] found that when the added DNA base sequence was A (A30) or C (C30), the synthesized nanoparticles were flower-like (named AuNF-A30 and AuNF-C30, respectively) (Figure 1A). However, the nanoparticles synthesized with T base (T30) were spherical in shape (AuNP-T30). Nanoparticles obtained with shorter DNA consisting of the 10-membered PolyG were also almost spherical. Furthermore, only spherical nanoparticles were formed without the addition of DNA or with the addition of salt alone. These findings suggest that DNA can act as an intermediary to control the shape of gold nanoparticles and that the shape of nanoparticles is sequence-dependent. The difference in binding affinity of different base pairs on the surface of Au nanoparticles is the primary reason for the different morphological nanostructures. T30 has a weak binding affinity to the surface of gold nanoparticles and adsorbs less DNA on the surface of gold nano-seeds (AuNS). In contrast, A30 or C30 can tightly bind to AuNS, inducing uneven growth and forming flower-like nanoparticles. Notably, when individual deoxynucleotide monophosphate adenosine (AMP) rather than DNA strands was incubated with AuNS, the resulting nanoparticles were almost spherical, whereas random 30-membered DNA sequences with a mixture of A, T, G, or C resulted in the formation of flower-like nanoparticles. Moreover, during the formation of nanocrystals, a part of DNA is buried in gold nanoflowers (AuNFs), while another part remains exposed outside the nanostructure, still retaining the hybridization function of DNA.

During crystal growth, different DNA sequences can influence the morphology of generated gold nanoparticles. DNA with a chain-like structure can guide the deposition of reduced gold on gold nano-seeds, directing the nanoparticles to form different shapes [6,48,49]. Gold nano-seeds and DNA templates are essential cores for the formation of gold nanocrystals (AuNCs) with various morphologies.

The shape of the nanoseed also impacts the final shape of the resulting nanostructure. In addition to nanospheres, nanoprisms and nanorods are commonly used as nanoseeds [62]. Instead of using gold spherical nanoparticles, Tan et al. [11] used gold nanoprism as a seed in the presence of T30, G20, C30, and A30 homologous zwitterions to investigate the mechanism of the morphological evolution of generated gold nanoparticles, which evolved into nonagon, hexagon, and six-pointed stars with rough surfaces (Figure 1B). The process involves two main stages, starting with a rapid increase in diameter, followed by an increase in thickness. The anisotropic nature of gold nanoprisms makes it easier to monitor their morphological evolution from the surface and edges compared to isotropic gold nanospheres. When AuNRs are used as seeds, different DNA sequences are employed to control their overgrowth, resulting in gold nanoparticles with diverse shapes ranging from nano-cells to nano-octahedrons or something in between (Figure 1C) [61]. The growth initiates from the ends of AuNRs. The smaller the diameter growth, the stronger the binding affinity of DNA to the gold surface. By adjusting the base composition of the DNA sequence or introducing phosphorothioate modifications in the DNA, the geometric and plasmonic properties of gold nanoparticles can be precisely controlled.

In summary, DNA can influence the deposition of Au precursors on AuNP through four main aspects: (1) the binding of DNA to Au precursors, (2) the dissociation of DNA from AuNP, (3) the density of DNA on the AuNP surface, and (4) the mobility of DNA on the AuNP surface. Although both A and C have a high binding affinity to Au, the lower mobility of A on the Au surface compared to C leads to Au deposition on the unbound sites of DNA, resulting in a rough surface. In contrast, the high mobility of C allows for the uniform deposition of Au, resulting in a smooth surface [11]. Moreover, several factors, including gold precursors, reducing agents, and surfactants, greatly influence the precise control of the morphology and plasmonic properties of nanocrystals during the synthesis process. When using polyethylene glycol (PEG) and sodium dodecyl sulfate (SDS) as surfactants, AuNCs could not be produced on the surface of DNA-AuNRs. However, the addition of polyvinylpyrrolidone (PVP), cetyltrimethylammonium bromide (CTAB), Tween 20, and Tween 80 allowed for the growth of nanobranched chains on the DNA-AuNR surface, forming various branched nanostructures (Figure 1D) [41]. Among them, Tween 80 exhibited stronger regulation and resulted in a more regular morphology of branched nanostructures. However, there is no clear explanation in this literature for the cause of this phenomenon.

**Figure 2:**
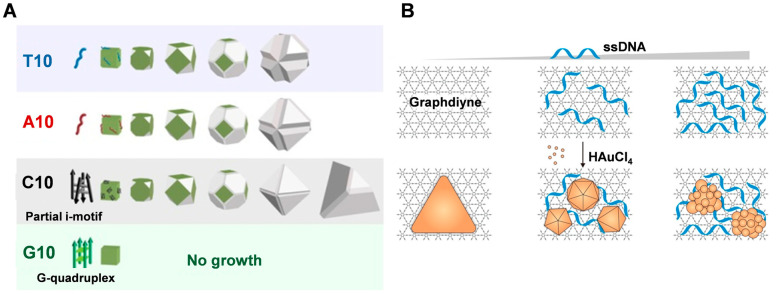
(**A**) DNA sequence-dependent morphological evolution of silver nanoparticles. Reproduced with permission [57]. Copyright 2014, American Chemical Society. (**B**) DNA-tailored Au growth on GD. Reproduced with permission [55]. Copyright 2020, American Chemical Society.

Different DNA sequences can affect the morphology and fluorescence properties of silver nanoparticles grown from silver nanoseeds (Figure 2A) [39,57]. The presence of 10-poly-oligo-A and -T directs the growth of AgNPs from nanocubic seeds to stellate octahedral AgNPs with different degrees of truncation. In the presence of poly-oligo-C10, truncated tetrahedral AgNPs are formed, while in the presence of oligo-G10, AgNPs maintain their original cubic shape and size. These shapes depend highly on the binding affinity of each base and the DNA secondary structure, favoring the stability of the Ag{111} facets. Sequence-dependent morphological control depends on three main aspects: the preferential binding capacity and stability of {111} facets over {100} facets, the affinity of DNA for Ag nanoparticles or Ag^+^ ions in solution, which affects the growth rate (e.g., A10 vs. T10), and the role of DNA secondary structure (e.g., formation of G-quadruplexes of the G or C of the i-motif). Furthermore, the sequence of DNA also affects the fluorescence properties of the resulting silver nanomaterials [39]. The G-rich template can produce a red emitter under acidic conditions that are susceptible to the G-quadruplex structure associated with the 5′ terminal guanine. C-rich sequences can produce red silver clusters under acidic or neutral conditions, and their emission is significantly regulated by the amount of cytosine at the 3′ end.

Graphdiyne (GD) is a novel two-dimensional carbon material that was used as a substrate to synthesize single-crystal gold nanostructures with tunable morphology at room temperature by Chen and co-workers [55]. The growth kinetics of AuNSs on GD was significantly faster compared to GO, which was attributed to the high reduction and adsorption capacity of GD (Figure 2B). Additionally, the introduction of single-stranded DNA resulted in the generation of polygonal and flower-like nanoparticles with tunable size and anisotropy, which can be attributed to the strong adsorption of DNA on the GD template altering the homogeneity of the interface. This provides a direct and versatile method for synthesizing Au nanostructures with tunable morphology and photonic properties.

### 3.2. dsDNA

In addition to using single-stranded DNA, double-stranded DNA can also be employed to engineer the controlled crystallization of gold and other metal nanomaterials [63,64]. Compared to single-stranded DNA, double-stranded DNA is more rigid and can remain upright within a certain number of bases on the surface of metal nanoparticles, enhancing its ability to orient the synthesis of AuNCs [65].

**Figure 3:**
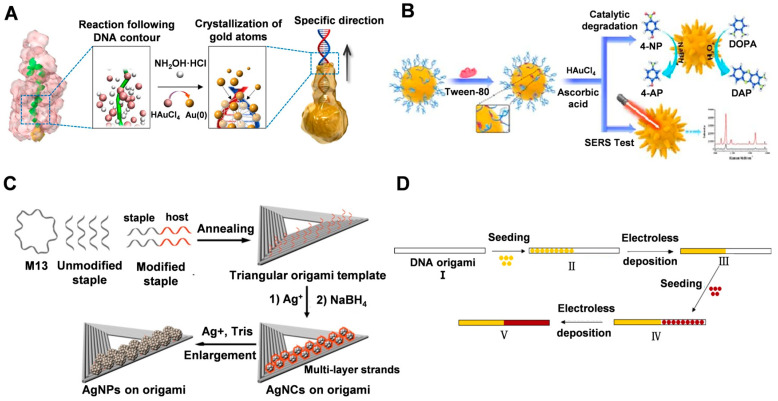
(**A**) Scheme showing DNA-directed crystallization of a AuNC. Reproduced with permission [66]. Copyright 2016, Springer Nature. (**B**) Schematic of the synthesis of sea urchin-shaped AuNCs for SERS analysis and catalytic degradation. Reproduced with permission [53]. Copyright 2021, IOP Publishing. (**C**) DNA-based nanotemplate directed in situ synthesis of silver nanoclusters. Reproduced with permission [58]. Copyright 2016, American Chemical Society. (**D**) The process used for making a heterogeneous metal junction on a DNA origami template. Reproduced with permission [56]. Copyright 2014, American Chemical Society.

Ma et al. [66] immobilized dsDNA on nanoseeds by modification, making them ideal molecular guides for the directional crystallization of nanocrystals. As shown in Figure 3A, double-stranded DNA molecules grafted on gold nanoseeds can regulate the crystallization process from the AuNS surface to the Au atoms at the ends of the DNA, which is driven by the distribution gradient of the gold precursor (HAuCl_4_). In bottom-up synthesis, it is easier to manipulate the crystallization of atoms in a guided direction when structurally controlling the morphology of nanomaterials, compared to using metalized molecular templates where the expansion of metal clusters only amplifies the initial shape of the template. This approach allows the formation of gold nanocrystals with different structures, such as asymmetric structures such as pegs, stars, and biconcave discs, as well as more complex jellyfish and flower-like structures, by using different arrangements to anchor the DNA to the gold nanoseeds [66]. It is worth noting that while the growth of DNA-directed branches can be controlled, the crystallization of the nanoseeds is driven by a thermodynamic tendency to reduce the surface energy, which is beyond external control. Thus, the relatively limited control of nanoseed growth does not allow 100% control of the morphology of the resulting AuNC structures [66]. Gao et al. [53] successfully synthesized sea urchin-like AuNCs with tunable plasmonic properties using a self-assembly technique of hybridized double-stranded polyadenine (dsPolyA) DNA (Figure 3B). The hybridized dsPolyA serves as a guide template with suitable rigidity and upright conformation, facilitating the formation of anisotropic multi-branched gold nanocrystals with the assistance of surfactants. The ratio of gold precursor to reducing agent and the DNA addition ratio both affect the density and distribution of the surface protrusions of the synthesized AuNCs.

### 3.3. DNA Origami

DNA origami is a technique that allows DNA molecules to be folded into specific shapes using complementary base-pairing. This method involves using DNA-origami structures as templates to direct the synthesis of metallic structures. These DNA origami structures can be used as templates to guide the synthesis of metallic structures with precise control of their size and shape. For example, DNA origami structures can be designed to be a specific shape, such as a triangle or square, and then used as a template to guide the synthesis of metal nanoparticles of that shape [47,54,67,68,69]. Ren et al. [54] used ribbon DNA origami nanostructures (RDN) as a template to assemble plasmonic gold metamaterials. Thiol DNA-functionalized AuNPs or gold nanorods (SH-DNA-AuNPs/SH-DNA-AuNRs) were mixed with five DNA strands in a single pot and assembled into high-quality one- and two-dimensional wires and lattices, resulting in enhanced Raman scattering [47].

Nanomaterials composed of other materials, such as silver, can be prepared using DNA origami as a template to obtain finely-structured nanostructures. In their research, Wang et al. [58] utilized single-stranded DNA with varying sequences as host strands for the site-specific synthesis of silver nanoclusters on the constructed DNA templates (as shown in Figure 3C). Their findings demonstrated, for the first time, that the rigid template affected the affinity of the reactant Ag^+^ to the host strand through the surface site resistance effect, resulting in the site-specific formation of silver nanocrystals (AgNCs) with specific fluorescence wavelengths. Furthermore, the excitation/emission properties of AgNCs could be regulated by adjusting the distance between the nucleation site and the template, the template conformation, and the position of the nucleation site on the template.

In addition to homogeneous structures, DNA origami can also be utilized for heterogeneous structural junctions. Uprety et al. [56] investigated the selective deposition of two different metals, copper and gold, on a DNA origami template that was designed and assembled to guide the deposition process. The resulting non-homogeneous Cu-Au junctions were formed through sequential seeding and deposition, as illustrated in Figure 3D.

In summary, the electrostatic interactions between metal ions and phosphate groups of DNA facilitate the homogeneous metallization of the DNA structure, allowing for the direct growth of inorganic nanocrystals on the DNA template. However, this approach lacks addressability and does not allow for the fabrication of arbitrary forms of inorganic nanostructures. Selective metallization at specific sites on DNA origami can address this limitation. Sequential selective metallization can provide the necessary microenvironment to confine and control the mineralization process, thus enabling the precise preparation of fine metal nanostructures.

## 4. Biomedical Applications

The biomedical applications of DNA-guided metallized nanomaterials are diverse and promising, with potential applications in biosensing, bioimaging, and therapy [70,71].

### 4.1. Biosensing

**Figure 4:**
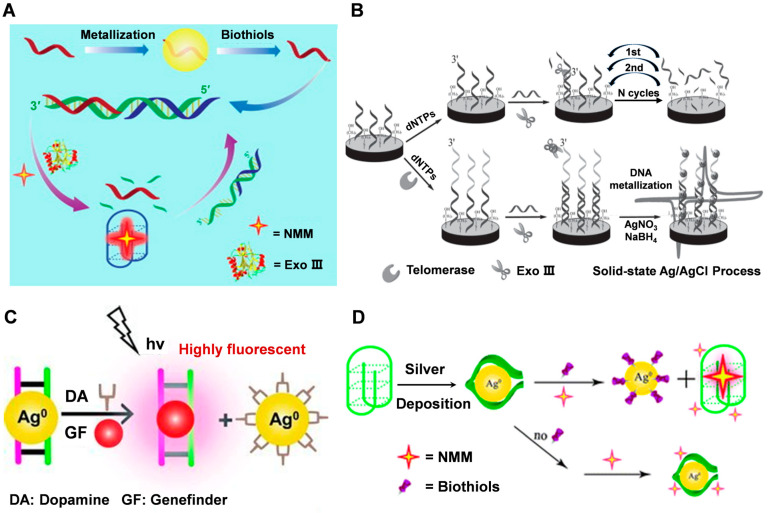
(**A**) Amplified detection of biothiols by coupling Exo III and DNA metallization. Reproduced with permission [59]. Copyright 2014, Elsevier. (**B**) DNA-metallization based signal amplification assay for human telomerase activity detection. Reproduced with permission [40]. Copyright 2014, Wiley-VCH. (**C**) The strategy for dopamine (DA) detection using DNA templated silver nanoparticles and intercalating dye genefinder (GF). Reproduced with permission [72]. Copyright 2011, Wiley-VCH. (**D**) The strategy for biothiol detection using DNA metallization engineered conformational switch of G-quadruplex DNA and intercalating dye NMM. Reproduced with permission [75]. Copyright 2012, The Royal Society of Chemistry.

DNA-guided metallized nanomaterials can also be used for biosensing applications, such as detecting biomolecules or pathogens in biological samples. By attaching DNA or antibodies to the surface of metal nanoparticles, specific interactions with target molecules can be detected using techniques such as surface plasmon resonance or electrochemistry. Chen et al. [59] utilized Exonuclease III (Exo III) biocatalysts and silver metallization of DNA to scale up biomercaptans at picomolar concentrations. The scale-up process relies primarily on the recovery of biomercaptans, the recovery of target DNA from silver deposits, and specific interactions between the quadchain and its binding ligands. DNA-silver nanohybrids are synthesized via NaBH_4_ reduction of AgNO_3_ (Figure 4A). Building upon this research, Wu et al. [40] developed a DNA metallization-based telomerase activity assay. The method utilizes a highly characteristic solid-state electrochemical process that utilizes DNA template deposition of silver nanoparticles as an electroactive label with enzyme-assisted suppression of background signals (Figure 4B). The ion exchange process is highly selective and limited to the DNA template, significantly improving sensing performance and reducing non-specific adsorption. Furthermore, this test does not require PCR amplification, thus avoiding related errors and contamination, and enabling a more reliable evaluation of telomerase activity in circulating tumor cells (CTCs).

DNA has a high affinity for silver ions, and these local cations can be reduced to form silver nanostructures that follow the profile of the DNA template. Thus, the formation of silver nanoparticles in the DNA scaffold would block the binding of ligands embedded in DNA and may also act as a fluorescent bursting agent when some ligands are embedded in the silver-adsorbed DNA. Lin et al. [72] first described the use of DNA-mediated silver nanostructures as a platform for simple, reliable, highly sensitive, and selective fluorescence-on detection of dopamine (DA). The method relies on large fluorescence enhancement through specific binding of the small molecule genefinder (GF) to dsDNA, which is released from the silver nanoparticles by DA (Figure 4C). Hao et al. [73] introduced multiple electroactive probes for the rapid detection of Cytokeratin fragment antigen 21-1 (CYFRA 21-1) DNA by surface-initiated reversible addition fragmentation chain transfer (SI-RAFT) polymerization and in situ DNA metallization as a signal amplification strategy, using C_3_H_4_O as a monomer. In the case of C_3_H_4_O as a monomer, SI-RAFT polymerization can bring a large number of aldehyde sites to the subsequent silver mirror reaction. The acrolein polymer acts as a reducing agent to reduce Ag^+^ to AgO, and then silver particles are deposited on the polymer backbone, which significantly amplifies the electrochemical signal. Gong et al. [74] used the DNA-guided growth of silver nanoclusters as a template to label catalytic and molecular beacons as an amplified biosensing platform for the detection of DNAzyme cofactors such as Pb^2+^ and L-histidine. The introduction of target cofactors triggers enhanced the fluorescence of AgNCs, thus providing an “on” fluorescence response to the target biomolecule. The proposed sensing system shows a highly sensitive response to the target cofactor by cyclic amplification using multiple enzymatic conversions of the DNAzyme. Chen et al. [75] presented the first method to implement a simple and label-free bio-thiol detection platform based on silver metallization of G-quadruplex DNA to control conformational switching. A simple, label-free, highly sensitive, and selective sensor was demonstrated for the detection of biothiols based on G-quadruplex conformational transitions designed by silver metallization. The method relies on a significant fluorescence enhancement resulting from a specific interaction between the NMM and the G-quadruplex, whose conformation is recovered after the release of the biothiols from the silver deposition (Figure 4D).

After using dsDNA to guide copper ions for metallization, they can also be used as detection probes [63]. Chen et al. [64] designed a new and simple strategy for highly sensitive and selective detection of Pb^2+^ using dsDNA-CuNPs as fluorescent probes. dsDNA can be used as an effective template to reduce Cu^2+^ by ascorbic acid to form CuNPs, and the formed dsDNA-CuNPs have superior fluorescence. Interestingly, it was found that Pb^2+^ could quench the fluorescence of dsDNA-CuNPs. Based on this phenomenon found in this work, a very simple and rapid method for Pb^2+^ monitoring was established.

### 4.2. Bioimaging

Similarly, DNA-guided metallized nanomaterials can also be used as contrast agents in biomedical imaging techniques such as magnetic resonance imaging (MRI) and computed tomography (CT). By attaching metal nanoparticles to DNA templates, contrast agents with high specificity and sensitivity can be designed for use in diagnostic imaging. Zhang et al. [41] produced sea cucumber-like gold nanocrystals (AuNC) with tunable plasmonic properties using DNA as a template. These sea cucumber-like AuNCs exhibit a broad absorption band in the near-infrared range (700–1100 nm) and possess good thermal stability, high photothermal conversion efficiency, and biocompatibility. They were utilized for in vitro CT imaging, dark field imaging, and photothermal therapy.

**Figure 5:**
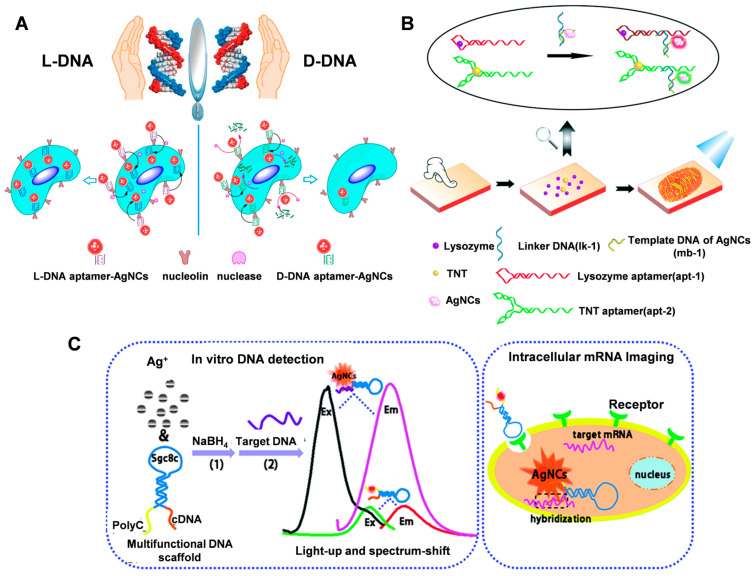
(**A**) Application of L-DNA or D-DNA-templated aptamer-AgNCs in cell-type-specific imaging. Reproduced with permission [79]. Copyright 2016, American Chemical Society. (**B**) Visualizing the LFPs using DNA-regulated AgNCs. Reproduced with permission [80]. Copyright 2016, The Royal Society of Chemistry. (**C**) The aptamer-functionalized AgNC-mediated in vitro DNA detection and intracellular mRNA imaging. Reproduced with permission [81]. Copyright 2014, The Royal Society of Chemistry.

Silver nanoclusters (AgNCs) prepared using DNA as a template have become an important tool for the development of biomarkers and molecular sensors because of their good fluorescence properties, excellent photostability, sub-nanometer size, and low cytotoxicity [76,77]. The fluorescent nature of silver nanoclusters is highly dependent on the DNA sequence and sensitive to the oligonucleotide environment, which gives them potential for genetic diagnostics. It was shown that strong base-Ag^+^ binding and the formation of stable supramolecular structures such as i-motifs and G-quadruplexes are two important prerequisites for the formation of bright photoluminescent AgNC [78]. Based on this principle, combining functional motifs of DNA (e.g., aptamers) with DNA templates for fluorescent DNA/AgNCs has been used for cell type-specific imaging. Han et al. [79] reported the first synthesis of aptamer-AgNCs using L-DNA as a template. Compared with natural D-DNA-templated AgNCs, L-DNA-templated aptamer-AgNCs have higher nuclease resistance (Figure 5A). This advantage makes the aptamer-AgNCs obtained by L-DNA templating more suitable for achieving cell type-specific imaging. Ran et al. [80] used multicolor C-rich DNA template AgNCs for the first time to simultaneously image the exogenous components of the latent fingerprint (LFP) and the LFP itself (Figure 5B). Visualization and detection of potential fingerprints are achieved by combining DNA-regulated AgNCs with molecular conjugates.

The versatility of DNA can also be exploited to combine different functional DNAs in a single structure for bioimaging. Li et al. [81] designed a multifunctional DNA scaffold to synthesize AgNCs for intracellular imaging of tumor-associated mRNAs (Figure 5C). The DNA scaffold contains three functional parts: SGC8c aptamer as a specific internalization part, fluorescent AgNC_S_ nucleating sequences, and complementary sequences (CDNAs) that can hybridize with target DNA or RNA to alter the fluorescent properties of AgNC_S_. In addition, DNA-AgNCs combined with other nanomaterials can be combined to design FRET-based fluorescent probes for the fluorescent labeling of folate receptors on cancer cells [76].

### 4.3. Therapy

**Figure 6:**
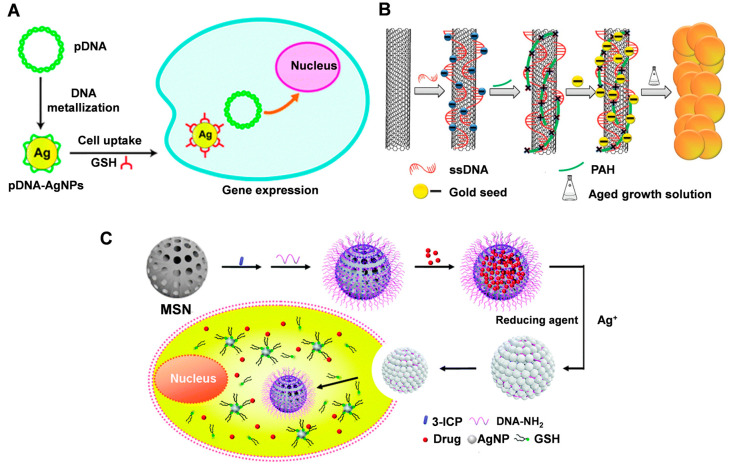
(**A**) Schematic illustration of pDNA-AgNP synthesis and gene delivery. Reproduced with permission [83]. Copyright 2013, The Royal Society of Chemistry. (**B**) The synthetic procedure of SWNTs-metal nanocomposite. Reproduced with permission [84]. Copyright 2012, American Chemical Society. (**C**) MSN gatekeepers designed for drug delivery, showing in situ growth and capping of AgNPs on the MSN surface via reduction of Ag^+^ with a DNA template and removal of the capped AgNPs by intracellular GSH to release the loaded drug. Reproduced with permission [85]. Copyright 2015, The Royal Society of Chemistry.

The use of DNA-guided metallized nanomaterials in therapy has the potential to improve efficacy and specificity. By attaching drugs to the surface of metal nanoparticles guided by DNA templates, targeted drug delivery to specific tissues or cells can be achieved [82]. Additionally, DNA-guided metallized nanomaterials can be designed to respond to specific environmental stimuli, such as changes in pH or temperature, leading to controlled drug release. In addition, the photothermal properties of metallic materials make them well-suited for photothermal therapy. Silver nanoparticles grown with DNA as a template are excellent platforms for gene delivery; DNA has a high affinity for silver ions and these local cations can be reduced to form silver nanostructures that follow the contours of the DNA template; the formation of silver nanoparticles (AgNP) in the DNA scaffold can spontaneously induce DNA bending and cohesion as well as negative charge shielding, which facilitates cellular internalization. Based on this, Tao et al. [83] reported a simple one-pot synthesis of plasmid DNA template silver nanoparticles (pDNA-AgNPs), which can be used as a platform for efficient gene delivery. The intracellular repair of plasmid DNA (pDNA) can be achieved through a glutathione (GSH)-mediated ligand exchange process, which can facilitate efficient gene delivery (Figure 6A). The ease of synthesis and low cytotoxicity of metallized pDNA structures compared to conventional gene carriers make them suitable biocompatible nanomaterials for biomedical applications.

The nanoparticles obtained by the metallization of DNA can be used in combination with other materials for photothermal therapy. Wang et al. [84] combined DNA-functionalized SWCNT with DNA-guided synthesis of noble metal (Ag or Au) nanoparticles to form nanocomposites by an in situ liquid-phase synthesis method. The nanocomposites also exhibited significantly enhanced photothermal cancer cell killing due to the strong surface plasmon resonance absorption generated by the gold shells grown on the nanotube surface (Figure 6B).

DNA-templated metallized materials can be used to act as gatekeepers for drug delivery systems. Mesoporous silica is often used to deliver drugs; however, its porous structure is prone to drug leakage. Liu et al. [85] proposed a method to construct silver nanoparticles (AgNP) on mesoporous silica nanospheres (MSN) through a DNA templating process. The DNA strands bound to the MSN surface can form AgNP to close the pores and reduce drug leakage (Figure 6C). Drug release is determined by specific Ag-S interactions. Subject to varying degrees of glutathione within the tumor environment, site-specific drug release can be achieved using a controlled exchange process mediated by GSH through ligand breakdown of AgNP. This interaction does not result in the formation of toxic -SH components, making it more biocompatible. Decorating MSNs with AgNPs via a DNA templating process provides a more labor-intensive yet cost-effective and robust method of nanocarrier construction, unlike traditional covalent or non-covalent strategies. This approach can also be extended to other DNA metallization nanomaterials.

Overall, these recent examples demonstrate the potential of DNA-guided metallized nanomaterials in various biomedical applications and provide a foundation for further research and development in this field.

## 5. Advantages and Challenges

The use of DNA templates in metallization has several advantages. First, DNA is a highly specific and versatile biomolecule that can be easily synthesized and modified to provide a variety of templates for different metals and applications. Second, DNA provides a high degree of control over the shape and size of the metal nanoparticles, which is critical for many applications, particularly in biomedicine. Thirdly, DNA is biocompatible and biodegradable, making it an attractive material for use in biomedical applications [86]. Finally, it is a simple and cost-effective method that can be easily scaled up for large-scale production and it offers a high degree of reproducibility and uniformity in the resulting nanoparticle [42].

There are also limitations in DNA-guided metallization. One major limitation is the requirement for specialized DNA sequences and metal ions, which can limit the range of possible applications. The DNA-guided metallization method may not be suitable for the synthesis of certain types of nanoparticles that require more complex chemical reactions or processing steps [87]. When DNA is used for the morphology control of nanomaterials, a common step is the functionalization of DNA on the surface of nanoparticles [88]. However, the current methods for regulating the location and number of DNA ligands on nanoparticles are either overly complex or not conducive to large-scale production. Furthermore, current synthesis methods typically require high temperatures, high pressure, and long reaction times with low yield. There are also issues regarding batch-to-batch inconsistency and problems with purity. Achieving consistent and pure nanomaterials remains a challenge. Therefore, new methods need to be developed to improve synthesis efficiency.

Finally, While DNA is generally considered non-toxic, the metal nanoparticles used in DNA-guided metallization may be toxic to cells and tissues [89]. Moreover, the toxicity of the nanomaterials may depend on their size, shape, and surface chemistry. It is therefore crucial to carefully evaluate the toxicity of DNA-guided metallized nanomaterials in vitro and in vivo before their use in biomedical applications [90]. In addition, DNA is prone to degradation and removal in biological environments. Therefore, more stable and reliable DNA modification methods need to be developed to better realize the biomedical applications of nanomaterials.

## 6. Prospectives

Although there have been significant advances in the field of DNA-guided metallized nanomaterials, there is still much research that needs to be conducted to advance the field. Emerging trends and new directions in the field of DNA-guided metallization of nanomaterials are focused on expanding the range of applications and developing multifunctional nanomaterials [91].

Current methods for synthesizing DNA-guided metallized nanomaterials are often time-consuming and produce small quantities of nanoparticles. There is a need for more efficient and scalable synthesis methods to enable the production of larger quantities of nanomaterials for use in biomedical applications. Advances in computer simulations and modeling have enabled the rational design of DNA-guided metallized nanomaterials with specific properties and functions. By simulating the interactions between DNA molecules and metal ions, researchers can predict the size, shape, and properties of the resulting nanoparticles and optimize their performance. The standardization of DNA-guided metallized nanomaterials is important to ensure reproducibility and comparability of results across different research groups. The development of standardized protocols for synthesis, characterization, and evaluation of DNA-guided metallized nanomaterials is needed to facilitate the translation of these materials into clinical applications.

There is a growing interest in developing DNA-guided metallized nanomaterials that have multiple functions, such as targeted drug delivery, imaging, and biosensing. Further research is needed to optimize the design of multifunctional nanomaterials and to evaluate their performance in vitro and in vivo. The biocompatibility of DNA-guided metallized nanomaterials needs to be thoroughly evaluated to ensure that they are safe for use in biomedical applications. More research is needed to determine the long-term effects of exposure to these nanomaterials and to develop strategies to mitigate any potential risks.

## 7. Conclusions

The field of DNA-guided metallization of nanomaterials has emerged as a promising strategy for the synthesis of nanomaterials with well-defined sizes, shapes, and compositions. This approach involves using DNA molecules as templates to guide the nucleation and growth of metal ions into nanoscale structures. In this review, we focus on the morphology control of gold and silver nanostructures using DNA, which can be modified on the surfaces of different nanomaterials through covalent or non-covalent binding [88]. Significant progress has been made in this field, with many successful demonstrations of DNA-guided metallization of a variety of nanomaterials, including gold, silver, platinum, and palladium nanoparticles, as well as nanowires and nanorods. The resulting nanomaterials exhibit unique properties and functionalities that make them attractive for a wide range of biomedical applications, including biosensing, drug delivery, and imaging.

The process of nanomaterials morphology control is influenced by several factors, including the shape of the nanoparticles, the density of DNA grafting, and the secondary structure of DNA. The shape of the nanoparticles strongly affects the interaction between the nanoparticles, which determines the orientation and assembly structure of the nanoparticles. The size, shape, and composition of precious metal nanoparticles (e.g., gold or silver) also affect their properties. When DNA oligonucleotides are in the single-stranded state, they are highly flexible and can be curled at will, while the double-stranded DNA structures formed after hybridization are very rigid. Hence, it is crucial to explore and control the multiple influencing factors in the future.

Further research is needed to advance the field of DNA-guided metallized nanomaterials and to fully realize their potential for biomedical applications. This includes the development of more efficient and scalable synthesis methods, the exploration of new biomedical applications, and the thorough evaluation of biocompatibility and safety. Additionally, it is still necessary to address how to translate the preparation method of DNA-guided metallization of nanomaterials into large-scale industrial production to meet the demands of biomedical applications.

Overall, DNA-guided metallization is a promising approach for the synthesis of metal nanoparticles and nanostructures with precise control over their size, shape, and composition. The use of DNA templates provides a powerful tool for the development of new materials with a wide range of biomedical applications.

## Figures and Tables

**Table 1:** DNA as director for nanomaterials metallization.

Seeds	Type of DNA Templates	Shape of Nanomaterials	References
AuNPs/AuNRs functionalized with thiol-DNA	Ribbon-like DNA origami nanostructures	1D AuNPs/AuNRs lines or 2D AuNPs lattices	[54]
30 nm AuNPs	Rectangular DNA origami	AuNP chains	[47]
Graphdiyne and HAuCl_4_	ssDNAs (A20)	Decahedrons, icosahedrons, or flower-like Au nanoparticles	[55]
Gold nanorods	ssDNA, dsDNA, hpDNA, and taDNA	Trepang-like AuNCs	[41]
AuNPs and Cu	Bar-shaped DNA origami	Cu-Au metal junction	[56]
Ag Nanocube	10-mer oligo-A, -T, -C	Edge-truncated octahedral, and truncated tetrahedral AgNPs	[57]
AgNO_3_	G-/C-Rich oligonucleotides	Silver clusters	[39]
AgNO_3_	Triangular DNA origami template	Ag nanoclusters	[58]
AgNO_3_	target DNA, its complementary sequence parts, and probe DNA G4)	Colloidal silver solution	[59]
AgNO_3_	Telomerase primer oligonucleotides	Silver NPs	[40]

## Data Availability

Data are contained within the article.
